# 

**Published:** 1894-01

**Authors:** 


### LIST OF CONTRIBUTORS.

ADAMI, J.
BELL, JOHN A.
BITTLES, E. E.
BLACK, D. EUGENE.
BOWKER, G. J.
BRYDEN, WILLIAMSON.
CARLTON, F. B.
CLEMENT, A. W.
CURTICE, COOPER.
DARLING, ANDREW.
DUNCAN, J. T.
ESHLEMAN, JOHN M.
FAUST, JOHN
FOX, D. F.
HADDEN, ALEX.
HEATH, A. S.
HEWLETT, R. T.
HUTCHINGS, ROBERT C.
LAW, JAMES.
LOVELAND, A. E.
MCWHINNE, H.
MATTSEN, W. H.
MEIKLE, JOHN.
MILLER, W. B. E.
MONSARRAT, W. T.
MORRIS, CLAUDE D.
NICHBERT, J. D.
PARAZOLS, M.
PARKER, JOHN M.
PIERCE, F. E.
REEFER, LEON N.
RUSSELL, F. L.
SPENCER, H. A.
STILES, CHAS. WARDELL.
TRUMBOWER, M. R.
TURNER, FRED. W.
TURNER, T. J.
VAN ES, L.
WATTLES, J. H.
WAUGH, JAS. A.
WENDE, JOHN.
WHITEHOUSE, A. W.
WILLIAMS, W. L.
WILLYOUNG, S. E.
WOLSTENHOLME, J. B.

### ILLUSTRATIONS, VOL. XV.

| Fig. | | Page. |
|---|---|---|
| 1. | A Study in Horse-Shoeing | 37, 38, 39, 206 |
| 2. | Anatomy of Taenia Crassicollis Rud | 86, 87, 88, 89 |
| 3. | The Emasculating Bot-Fly | 203 |
| 4. | Anatomy of the Large American Fluke, | 226, 227, 229, 231, 233, 236, 237, 239, 300, 304, |
| 5. | The Point system of Judging | 350 |

### INDEX VOLUME XV.

### A

PAGE
Anatomy of American Fluke, C. W. Stiles........................161, 225, 299
Army Veterinary Bill.......................................... 217
American Veterinary College................................... 264
Act to Establish Live Stock Commission, Pennsylvania.......... 463
Adami, J. G., M. A., M. D..................................... 444
Army Legislation, Report of Committee on...................... 400
Abnormal results following Dehorning Cattle................... 449
Anatomy of the Large American Fluke.......................407, 457
Age, The Effect of, on the Organs of the Horse................ 438
Anthrax, Value of Prophylactic Treatment...................... 440
Abdominal Hernia, successfully treated........................ 447

### B

Bot-fly—on the emasculating.................................. 200
Bell, John A., Nails in Feet.................................. 119
Bowker, G. J., Osteo-Porosis.................................. 9
Black, D. E., Iatrol as an Antiseptic......................... 35
Bryden, Williamson, Horse Shoeing.........................36, 206
Quittor....................................................... 43
Tuberculosis.................................................. 207
Bittles, E. E., V. S.......................................... 447
Blood serum Therapeutics...................................... 418

### C

Chronic Bright's Disease, Andrew Darling...................... 10
Curtice Cooper, Tuberculin in Practice........................ 11
Choking and the Treatment, John Meikle........................ 121
Carlton, F. B., Homeopathy.................................... 179
Communication, Ohio Veterinary College........................ 218
Climate, Influence of, W. L. Williams......................... 313
Current Literature for Veterinarians.......................48, 124
Change of Management.......................................... 269
Chicago Veterinary College.................................... 262
California State Veterinary Medical Association...........58, 272, 357
Civil Service Examination..................................... 423
Clement, A. W., V. S.......................................... 455
College Notes................................................. 476
Constitution and By-laws, U. S. V. M. A....................... 396
Comparative Pathology......................................... 444

### D

Darling, Andrew, Chronic Bright's Disease.... ........................ 10
Dehorning Cattle, A few Abnormal Results following.......... .......... 449
Diseases, Report of Committee on.............. .......... .. ...... 392
Dislocation between the second and third Vertebrae in the Horse............ 455
Dog in Human Society, Alex Hadden.............................. .. 17
Duncan, J. T.............................. ...... ........... 438

### E

Editorial—Current Literature for Veterinarians........................48, 124
Army Veterinary Bill................ ....... ................ 217
United States Veterinary Medical Association.......... ....... 267
Veterinary Legislation...................................... 268
Veterinary Education........... ............................ 269
Change of Management.... ....................... .......... 269
McKillip Veterinary College........ ....................... 353
Journal of Comparative Medicine and Veterinary Archives....... 472
Roads to Veterinary Education................. ....... .... 472
Veterinary Education, Roads to............................. 472
Blood Serum Therapeutics.......... ....................... 418
Embargo on American Cattle................................. 418
Tuberculosis....... .......... .......... ............... 419
How might we best spend the Winter Sessions of our Association?. 420
Effect of Age on the Organs of the Horse..................... 438
Eshleman, John M., Haemoglobinuria................................ 115
Complicated Wound of Leg........................ 122
Equine Ovariotomy, Jas. A. Waugh........................... ... ... 195
Erie County Veterinary Medical Society................................ 279

### F

Fluke, Anatomy of American................ ................161, 225, 299
Fasciola Magna, C. W. Stiles ...... .................. ... ........ 161
Fox, D. F., Liver Diseases........... ............................ 340
Faust, John, V. S.......................... ............ ......... 440

### G

German Veterinary Medical Association............................134, 271
Graduates 1894 :
Ontario Veterinary College............................ ........ .. 254
Kansas City Veterinary College........................... ....... 259
Ohio Veterinary College............................ ................ 260
New York College of Veterinary Surgeons...... ........ ......... 261
Chicago Veterinary College........................... .. .. .... 262
American Veterinary College.................................... 264

### H

Hadden, Alex., The Dog in Human Society...... ...................... 17
Horse-shoeing, A Study in, W. Bryden.... .........................36, 206
Haemoglobinuria, J. M. Eshleman.......... .......... ... ........ .. 115
Hutchings, Robert C., Obituary. ................................... 123

Homeopathy, F. B. Carlton .......... 179
Heath, A. S., Tuberculosis .......... 204
Hewlett, R. T., Tetanus .......... 209
Harger, S. J. J., Neurectomy as a Practical Operation .......... 365
Hypodermic Injections, Treatment of Ossifiic Disease of the Joints by .......... 450

I

Influenza, W. B. E. Miller .......... 28
Iatrol as an Antiseptic, D. E. Black .......... 35
Intelligence and Education, Report of Committee on .......... 382
Incorporation, Report of Committee on .......... 398

J

Judging, Point System of .......... 348

K

Kangaroos, Notes on, A. W. Whitehouse .......... 187
Kansas City Veterinary College .......... 259
Keystone Veterinary Medical Association .......... 56, 134, 436

L

Leg, Complicated Wound of, J. M. Eshleman .......... 122
Loveland, A. E., Taenia Crassicollis Rud .......... 67
Law, James, Unsuspected Poisoning .......... 101
Liver, Diseases of, D. F. Fox .......... 340

Mc

McWhinne, H., Carbuncle .......... 244
McKillip Veterinary College .......... 353

M

Miller, W. B. E., Influenza .......... 28
Mattsen, W. H., Osteo Porosis .......... 41
Tubercular Meningitis .......... 47
Morris, Claude D., Professional Etiuquette .......... 109
Meikle, John, Belladonna in Choking .......... 121
Monsarrat, W. T., Prolonged Pregnancy .......... 216
Maine Veterinary Medical Association .......... 355
Maryland State Veterinary Medical Association .......... 359
Montreal Veterinary Medical Association .......... 57
Massachusetts Veterinary Association .......... 128, 276
Massachusetts, Tuberculosis .......... 466, 469
Manitoba Veterinary Association .......... 471
Missouri Veterinary Medical Association .......... 435

N

New York College of Veterinary Surgeons .......... 261
Nails in Feet, John A. Bell .......... 119
New Remedies in Veterinary Medicine, F. W. Turner ..........
Neurectomy as a Practical Operation .......... 365

Nomenclature, Report of Committee on... 402
New Jersey, Veterinary Medical Association of ... 436
Nichbert, Dr. J. D ... 449

O

Ohio Veterinary College... 260
Ontario Veterinary College... 254
Ontario Veterinary Association... 65, 159
Ovariotomy, S. E. Willyoung... 197
Osteo-Porosis, G. J. Bowker... 9
Osteo-Porosis, W. H. Mattsen ... 41
Obituary, Robert C. Hutchings... 123
Obituary, John S. Saunders... 292
Ossific Disease of the Joints, Treatment of, by Hypodermic Injections... 450
Ontario Veterinary College... 483
Obituary... 484

P

Purpura Hemorrhagica, H. A. Spencer... 245
Parker, John M., Tuberculosis ... 182
Parazols, M., Stagges... 40
Pierce, F. E., Parturient Apoplexy... 248
Point System of Judging ... 348
Professional Etiquette, C. D. Morris... 109
Pyo-Septhamie in Foals, John Wende ... 116
Prolonged Pregnancy, W. T. Monsarrat... 216
Publication Committee ... 391
Pennsylvania, Act to Establish Live Stock Commission... 463

Q

Quittor, W. Bryden... 43

R

Reefer, Leon N., Ruptured Bladder... 27
Ruptured Bladder, Leon N. Reefer... 27
Regulations, Massachusetts... 469
Report of Committee on Intelligence and Education... 382
" Publication Committee... 391
" Committee on Diseases ... 392
" " " Revision of Constitution and By-Laws... 396
" " " Incorporation ... 398
" " " Army Legislation... 401
" " " Nomenclature ... 402
" Secretary ... 404
Russell, F. L., V. S... 453

S

Staggers, Treatment of, M. Parazols... 40
Stiles, Charles Wardell, Anatomy of American Fluke... 161, 225, 299

Spencer, H. A., Purpura Hemorrhagica ........ 245
Secretary's Report U. S. V. M. A ........ 404
Stiles, Chas. Wardell, Ph. D ........ 407, 457
Society Proceedings:
Erie County Veterinary Medical Society ........ 279
Maine Veterinary Medical Association ........ 355
Maryland State Veterinary Medical Association ........ 359
Veterinary Medical Association of New Jersey ........ 55
Keystone Veterinary Medical Association ........ 56, 134, 436
Montreal Veterinary Medical Association ........ 57
California State Veterinary Medical Association ........ 58, 272, 357
Ontario Veterinary Association ........ 65, 159
New York Veterinary Societies ........ 127
Pennsylvania State Veterinary Medical Association ........ 127, 280
Massachusetts Veterinary Association ........ 128, 276
German Veterinary Medical Association, New York City ........ 134, 271
New York State Veterinary Medical Society ........ 136
United States Veterinary Medical Association ........ 154, 292
Veterinary Medical Association of New York County ........ 155, 221, 250
North Dakota Medical Association ........ 157
Veterinary Medical Association of Oneida County ........ 222
Ohio State Veterinary Medical Association ........ 223
Iowa State Veterinary Medical Association ........ 485
Keystone Veterinary Medical Association ........ 488
Illinois State Veterinary Medical Association ........ 489
Society for the Study of Comparative Psychology ........ 490
United States Veterinary Medical Association, 31st Annual Meeting, 424
Missouri State Veterinary Medical Association ........ 435
United States Veterinary Medical Association, Reports of Committees ........ 382, 391, 392, 396, 398, 400, 402

### T

Tetanus R. T. Hewlett ........ 209
Turner, F. W., New Remedies in Veterinary Medicine ........ 1
Tuberculin in Practice, Cooper Curtice ........ 11
Tubercular Meningitis, W. H. Mattsen ........ 47
Taenia Crassicollis Rud, A. E. Loveland ........ 67
Tuberculosis, J. M. Parker ........ 182
A. S. Heath ........ 204
W. Bryden ........ 207
Trumbower, M. R., Tuberculosis ........ 335
Tuberculosis, Pennsylvania ........ 463
Tuberculosis, Massachusetts ........ 466, 469
Tuberculosis ........ 453
Tuberculosis ........ 419, 453

### U

Unsuspected Poisoning by Sterilized Meat, James Law ........ 101
United States Veterinary Medical Association ........ 154, 267, 293

### V

Van Es, L., Veterinary Opthalmology.......... ...................... 90
Vomition. J. B. Wolstenholme........................................ .. 214
Veterinary Legislation.............................................. 268
Veterinary Education .. ................. .............. .. ... ..... 266
Veterinary Medical Association of New Jersey.......................... 55
Veterinary Association of Manitoba. ........ .......... ............ 471
Veterinary News........... .............. .... . ................. 475
Value of Prophylactic Treatment in Anthrax ...................... ...... 440
Veterinary Medical Association of New Jersey........... ...... ........ 436

### W

Wende, John, Pyo-Septhamie.......... ....................... . . .. 116
Whitehouse, A. W., Notes on Kangaroos...... ....................... 187
Waugh, Jas. A., Equine Ovariotomy.................................. 195
Willyoung, S. E., Ovariotomy................. ...... .. .......... 197
Wolstenholme, J. B., Vomition...... ...... ........................ 214
Williams, W. L., Influence of Climate.......... ...................... 313
Walley, Prof. Thomas, Obituary................. ......... ....... 484
Wattles, Junius H., D. V. S... ........ ... ....................... 450
Winter Sessions of Our Association.................................. 420

